# Transcriptomic and chromatin accessibility dynamics of porcine alveolar macrophages in exposure to fumonisin B1

**DOI:** 10.3389/fcell.2022.876247

**Published:** 2022-10-18

**Authors:** Jian Jin, Jiayao Jiang, Zhengchang Wu, Ruihua Huang, Mingan Sun, Wenbin Bao

**Affiliations:** ^1^ College of Animal Science and Technology, Yangzhou University, Yangzhou, China; ^2^ Institute of Comparative Medicine, College of Veterinary Medicine, Yangzhou University, Yangzhou, China; ^3^ College of Animal Science and Technology, Nanjing Agricultural University, Nanjing, China

**Keywords:** fumonisin, transcriptomic, chromatin accessibility, pigs, macrophages

## Introduction

Fumonisins are a class of water-soluble secondary metabolite mainly produced by *Fusarium moniliforme* and *Fusarium rotundus*, which can contaminate a variety of foods and their products and severely affect agricultural and animal husbandry production (Scott, 1993). Fumonisin B1 (FB1) is a major component of fumonisin compounds and occupies an important position in the toxic effects of fumonisin (Lino et al., 2007; Stockmann-Juvala and Savolainen, 2008; Chen et al., 2020). FB1 is frequently found in corn-based foods, and is a key contaminant in a large number of food products throughout the world (Marina Martins et al., 2008; Domijan, 2012). The multiple toxic effects triggered by the exposure to FB1 in many animal species make FB1 contamination a severe public health problem (Hussein and Brasel, 2001; Wild and Gong, 2010). The high contamination rate and high detection cost of FB1 make it difficult to completely eliminate the risk of FB1 intake by livestock and poultry through feed (Ren et al., 2017). Clinically, long-term intake of food contaminated with fumonisin can increase the risk of esophageal cancer and cardiovascular disease in human, and cause nephrotoxicity, hepatotoxicity, neurotoxicity, and intestinal barrier dysfunction in different mammals, while ingestion of FB1-contaminated diets in pigs can specifically present with hydrothorax and pulmonary edema (Gbore and Egbunike, 2008; Scott, 2012; Stoev et al., 2012; Kamle et al., 2019; Régnier et al., 2019; Tardieu et al., 2019). Because pigs are an excellent model for cardiovascular and other diseases in humans, the mechanism for FB1 toxicosis in pigs must be characterized to permit assessment of its potential toxicity in human populations. At present, there are few studies on FB1-induced immunotoxicity, and studying the molecular mechanism of cellular action of FB1 toxicity will help to develop new prevention and control strategies for FB1.

The development and wide application of various omics approaches have greatly boosted different fields of biological and biomedical studies. Among them, RNA sequencing (RNA-seq) and Assay for Transposase-Accessible Chromatin with high-throughput sequencing (ATAC-seq) techniques are particularly powerful in genome-wide transcriptomic and regulatory profiling (Ayturk, 2019; Shashikant and Ettensohn, 2019). RNA-seq can be used to profile the abundance of messenger RNAs (mRNAs) which have protein coding potentials, and various types of non-coding RNAs (ncRNAs) including microRNAs (miRNAs), long non-coding RNAs (lncRNAs), and circular RNAs (circRNAs) which have distinct structural properties and regulatory functions (Ponting et al., 2009; Lindberg and Lundeberg, 2010; Lappalainen et al., 2013; Booton and Lindsay, 2014; Xu and Xie, 2018). The accessibility of chromatin affects the binding of transcription factor and activity of regulatory elements (e.g., promoters and enhancers), which regulates gene transcription (Tsompana and Buck, 2014; Lambert et al., 2018). While RNA-Seq is useful for transcriptomic profiling, recently developed ATAC-seq technique enables genome-wide profiling of chromatin accessibility landscape which facilitate the revealing of the regulatory mechanism (Buenrostro et al., 2013; Klemm et al., 2019). Notably, the integration of RNA-seq and ATAC-seq techniques determines both the transcriptional and regulatory landscapes, which can facilitate the revealing of regulatory mechanism between gene expression and chromatin accessibility, and identification of the underlying key transcription factors and regulatory networks.

**FIGURE 1:**
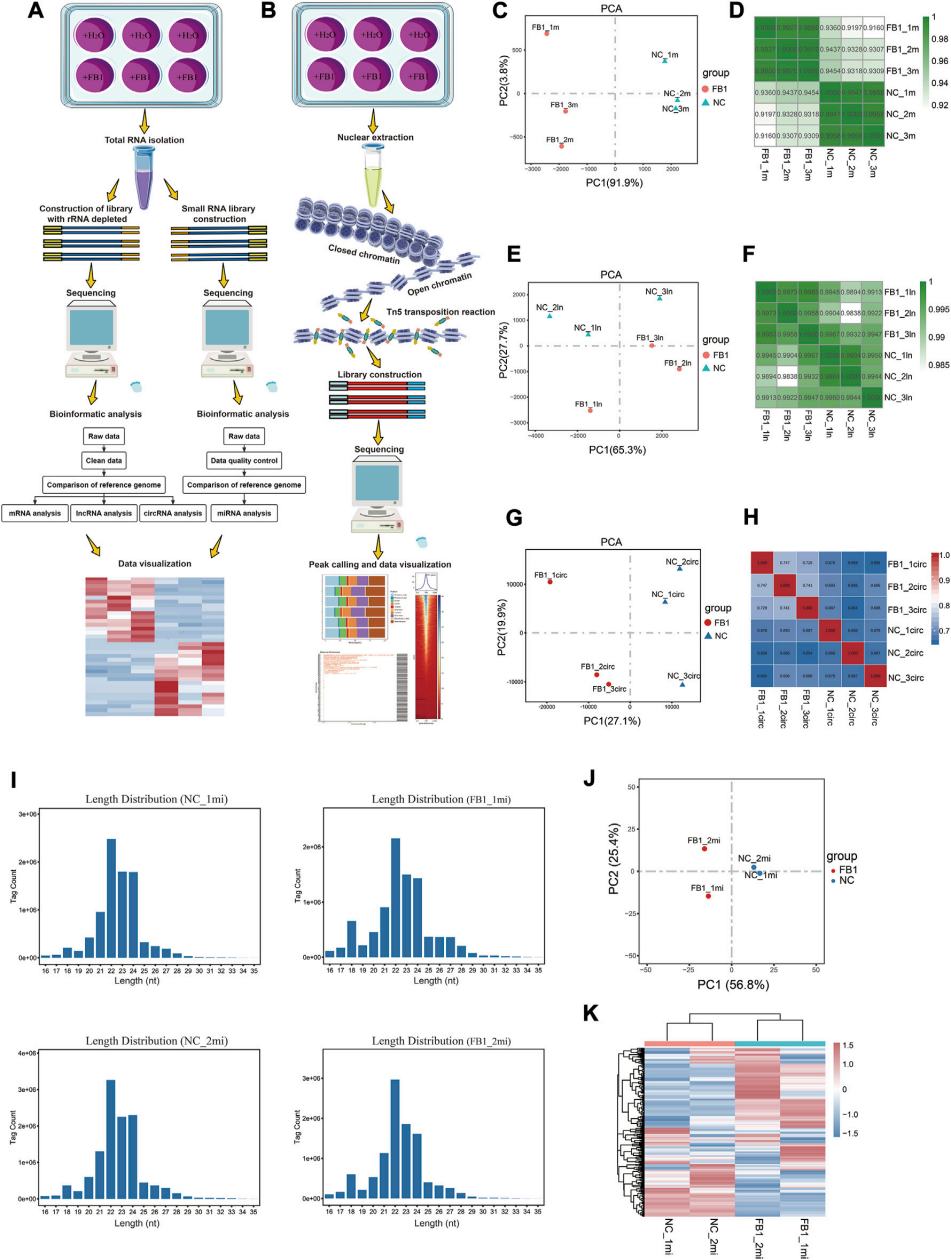
Schematic diagram of experimental design, data analysis workflow, and RNA-seq data quality metrics. These flow charts summarize the experimental and bioinformatic analysis procedures related to RNA-seq **(A)** and ATAC-seq **(B)**, respectively. The involved procedures, including those related to sample treatment and collection, sequencing library construction and bioinformatic analysis, are demonstrated. The figures visualize the Principal Component Analysis (PCA) and pair-wise correlation analysis results for different samples based on the expression profiles of mRNA **(C,D)**, lncRNA **(E,F)** and circRNA **(G,H)**, respectively. For the PCA plots, the *x*-axis and *y*-axis represent PC1 and PC2, respectively **(C,E,G)**. Regarding the pair-wise correlation analyses, Pearson’s was calculated and visualized by color gradients in the heatmaps **(D,F,H)**. **(I)** Distribution of miRNA sequencing read length for each sample. **(J)** Principal component analysis of miRNA. The *x*-axis and *y*-axis represent PC1 and PC2, respectively. **(K)** Visual heatmap analysis of miRNA expression profiles. The amount of miRNA expression is indicated by color gradients, with red indicating higher expression and blue indicating lower expression.

To understand the transcriptional and regulatory mechanisms underlying the cytotoxic effect of FB1, we applied RNA-seq for transcriptomic profiling of mRNA, lncRNA, circRNA and miRNA in porcine alveolar macrophages during FB1 exposure (Figure 1A), which generated a total of 468,818,429 high-quality reads (Supplementary Tables S2,S3). We also applied ATAC-seq to determine the genome-wide chromatin accessibility alterations upon FB1 exposure (Figure 1B), yielding a total of 467,539,462 high-quality reads (Supplementary Table S4). These data may facilitate the identification of the key genes and signaling pathways contributing to cellular response to FB1 exposure.

## Materials and methods

### Cell culture, treatment and collection

The cell viability of porcine alveolar macrophages (3D4/21) (ATCC, CRL-2843) treated with different concentrations (0, 10, 20, 30, 40, 50, and 60 μg/ml) of FB1 at different culture time points (24, 48, and 72 h) was measured on a Tecan Infinit 200 microplate reader (Tecan) platform using the Cell CountingKit-8 (CCK-8) kit (Dojindo, Shanghai, China), and finally 50 μg/ml FB1 was induced for 24 h as the optimal treatment concentration and action time to investigate the cytotoxicity of FB1 on 3D4/21 cells. Porcine alveolar macrophages were seeded in 6-well plates at a density of 5 × 10^5^ cells/mL and cultured in a 5% CO_2_ incubator at 37°C for 24 h. FB1 at a final concentration of 50 μg/ml was added to the culture medium of the experimental wells, and the same amount of enzyme-free water was added to the culture medium of the control wells. After 24 h of FB1 treatment of cells and control cell culture, cells were collected for RNA-seq and ATAC-seq, respectively. Three FB1 treated samples and three control samples were collected for strand-specific library construction (lncRNAs, mRNAs, circRNAs) of ribosomes depleted for RNA-seq (Supplementary Table S1). Two FB1 treated samples and two control samples were also collected for small RNA library construction (miRNA) (Supplementary Table S1). At the same time, three FB1 treated samples and three control samples were collected for ATAC-seq analysis (Supplementary Table S1).

### rRNA-depleted RNA-seq library construction

Total RNA was extracted from the experimental samples using the Trizol (Invitrogen, Carlsbad, CA, United States ) kit according to the instructions, RNA purity and concentration were preliminarily detected using a NanoDrop2000 spectrophotometer (Thermo Scientific, MA, United States ), and RNA integrity was accurately quantified using an Agilent 2100 (Agilent Technologies, CA, United States ) bioanalyzer. Then, we remove the rRNAs from the total RNA of the sample, retain mRNAs and ncRNAs, reverse transcribe the obtained RNA, purify the cDNA fragment using QiaQuick PCR kit (Qiagen, Venlo, Holland), repair the end, add PolyA, add sequencing linker, degrade the product by UNG (Uracil-N-Glycosylase) enzyme and amplify the product by PCR, and sequence the library by Illumina HiSeqTM4000.

### Small RNA library construction

Total RNA was extracted from the experimental samples using the Trizol (Invitrogen, Carlsbad, CA, United States ) kit according to the instructions. The RNA of the size of 18–30 nt was enriched by polyacrylamide gel electrophoresis (PAGE). The 3′ adapter and 5′ adapter were connected respectively, and the small RNA connected with the two adapters was reverse transcribed. The bands of 140–160 bp in size were amplified by PCR and recovered and purified to complete the library construction. The constructed library was quality controlled using an Agilent 2100 (Agilent Technologies, CA, United States ) and sequenced by Illumina HiSeqTM2500.

### RNA-seq data analysis

We used fastp v0.18.0 (Chen et al., 2018) for quality control and data filtering of raw reads from rRNA-depleted library, to remove reads containing adapters, with a N-containing proportion greater than 10%, with all A bases, or with bases with Q-value ≤20 accounting for more than 50% of the whole reads. The processed reads were aligned to the pig reference genome (release Sscrofa11.1) using HISAT2 v2.1.0 (Kim et al., 2015). To examine the relationship among different samples, Principal component analysis (PCA) was performed using R package gmodels (https://CRAN.R-project.org/package=gmodels). Differentially expressed genes were identified by DESeq2 (Love et al., 2014), with cut-off: FDR<0.05 and |log2(fold change)|≥1. The raw data of small RNA library was filtered to remove low quality reads containing more than one low quality (Q-value≤20) base or containing unknown nucleotides (N) in the data, to filter out reads without 3 ′adapters, to filter out reads containing 5′ adapters, and to filter out reads containing poly A. All clean tags were searched to identify known porcine miRNAs (exist miRNAs) using the miRbase database (release 22) (Griffiths-Jones et al., 2006).

### ATAC-seq library construction

The cell samples were collected and the nuclei were extracted, and the transposable mixture containing Tn5 transposase was added to the nuclear suspension for transposable reaction. Tn5 transposase entered the nucleus and preferentially cleaved exposed DNA in the open region of chromatin, while ligating specific sequencing adaptor. The DNA fragments ligated with adaptors were amplified by PCR, and the amplified PCR products were purified with the MinElute PCR Purification Kit (QIAGEN, Shanghai, China) and sequenced by Gene Denovo Biotechnology Co., Ltd. (Guangzhou, China).

### ATAC-seq data analysis

Data quality control was performed on ATAC-seq raw reads obtained from the sequencer before information analysis, and low-quality reads containing adapter reads, reads containing more than 10% unknown nucleotides (N), and low-quality reads containing more than 50% low quality (Q-value≤20) bases were removed to obtain high quality clean reads. The processed reads were aligned to the pig reference genome (release Sscrofa11.1) using Bowtie2 v2.2.8 (Langmead and Salzberg, 2012), with reads aligned to mitochondrial genome discarded, and reads that are uniquely aligned were used for subsequent analysis. The distribution map of insert fragments of each sample was drawn by ATACseqQC (Ou et al., 2018). DeepTools (Ramírez et al., 2016) was used to visualize the read distribution flanking transcription start sites (TSSs). Peak calling was performed using MACS v2.1.2 (Zhang et al., 2008) with a threshold of q-value < 0.05. Only the common peaks among replicates (with overlap of more than 50%) were retained for analysis. Peak annotation was performed using ChIPseeker v1.16.1 (Yu et al., 2015).

## Technical validation

### RNA-seq quality verification and data evaluation

For rRNA-depleted RNA-seq data, we confirmed that all samples are of similar sequencing depth, and the raw data are of good quality with >95% of bases pass the Q20 threshold. All these samples achieved alignment efficiency of higher than 91% (Supplementary Table S2). PCA and Pearson correlation analyses were used to understand consistency among replicated samples. PCA visualization according to the expression of mRNA, lncRNA, or circRNA revealed that these samples can be well clustered as two groups before and after exposure to FB1 (Figures 1C,E,G). The correlation coefficient among replicates was greater than 0.91 based on mRNA expression (Figure 1D), 0.98 based on lncRNA expression (Figure 1F), and 0.65 based on circRNA expression (Figure 1H). Together, these results indicate these data are of good quality and well replicated, therefore can be used for further analysis.

For miRNA-seq data, after quality control and pre-processing, more than 99% of the raw reads are retained as clean reads, and the proportions of clean tags of the small RNAs exceeded 98% (Supplementary Table S3). As expected, the lengths of most tags were distributed between 21 and 24 nt (Figure 1I), which was in consistent with the biological characteristics of small RNAs. PCA visualization showed that the samples were well clustered by groups before and after exposure to FB1, and the intra-group reproducibility of sequencing data was good (Figure 1J). In order to examine the miRNA expression patterns in different samples, the expression profiles of different miRNAs were further visualized as heatmap, which also suggest that our data are well replicated regarding miRNA expression profiles (Figure 1K).

### ATAC-seq quality verification and data evaluation

**FIGURE 2:**
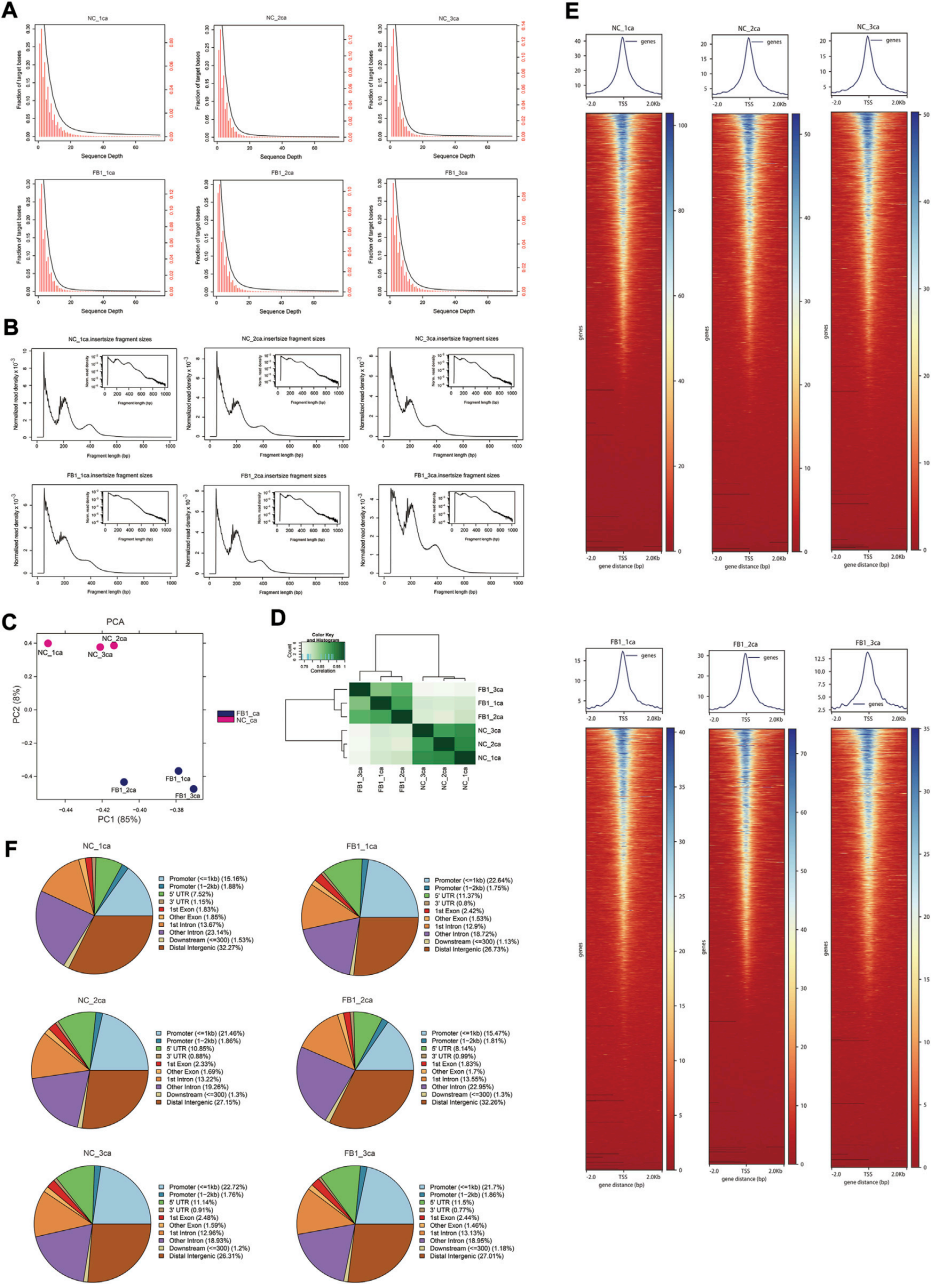
ATAC-seq data quality control and characteristics. **(A)** Distribution of sequencing depth across the genome. The *x*-axis is the sequencing depth. The *y*-axis on the left (in black color) corresponds to the black cumulative curve representing the fraction of genomic regions above corresponding sequencing depth, while the *y*-axis on the right (in red color) corresponds to red histogram representing the proportion of genomic regions corresponding to different sequencing depths. **(B)** Distribution of insertion fragment size of each sample. The *x*-axis represents the fragment size, and *y*-axis indicate the normalized read density. The small image in the upper right corner reflects the same information as the big image, except that its *y*-axis is log10-scaled. **(C)** PCA visualization of ATAC-seq samples. The *x*-axis and *y*-axis represent PC1 and PC2, respectively. **(D)** Heatmap showing the pair-wise correlation of ATAC-seq samples. Pearson’s r was calculated based on the ATAC-seq signal along the genome and visualized by color gradients in the heatmap. **(E)** Heatmaps (bottom) and averaged curves (top) showing the read distribution around TSSs based on ATAC-seq data of different samples. **(F)** Pie plots show the genomic distribution for the ATAC-seq peaks called for each sample. The categories for different genomic regions are indicated by different colors, and the percentages for each category are labelled in the brackets.

After quality control of ATAC-seq data, clean reads were mapped to the pig reference genome (Supplementary Table S4). The genomic distribution of uniquely aligned sequences was analyzed, and the read depth distributions across the genome were examined (Figure 2A). The chromatin accessibility fragments of the samples showed a size period corresponding to the integral multiple of nucleosomes, with the main peak between 10 and 100 bp was mainly the open region without nucleosome binding, and the small peaks around 200 bp and 400 bp being a DNA fragment bound to one or two nucleosomes respectively (Figure 2B). In addition, PCA analysis indicated that the samples were well clustered by their groups (Figure 2C). The pairwise Pearson’s correlation was calculated based on the read signals on the combined ATAC-seq peaks, which further indicated that the replicates from the same group resemble each other (Figure 2D). We visualized the signal distribution around the TSS and as expected, the ATAC-seq reads were strongly enriched around the TSS (Figure 2E), indicating that chromatin accessible regions could be successfully detected by ATAC-seq. Further examination of the genomic distribution of identified peaks indicate that only approximately 22% of them located in promoter regions, while remaining peaks are within exons, introns, and distal intergenic region—notably more than 50% of them fell into intronic and distal intergenic regions which are putative enhancers (Figure 2F).

## Conclusion

In summary, using the porcine alveolar macrophage cell line (3D4/21) as model, we applied both rRNA-depleted RNA sequencing (RNA-seq) and small RNA-seq to analyze the genome-wide transcriptional alterations of mRNA, lncRNA, circRNA and miRNA before and after exposure to FB1. To further reveal the underlying regulatory mechanism, we applied Assay for Transposase-Accessible Chromatin with high-throughput sequencing (ATAC-seq) to determine the genome-wide chromatin accessibility alterations in response to FB1-induced cytotoxicity. We anticipate that this dataset will serve as valuable resource for clarifying the transcriptional and regulatory mechanism underlying the immunotoxicity of FB1, and facilitate the identification of the key genes and signaling pathways contributing to mammalian cells response to FB1 exposure.

## Data Availability

All the raw and processed data generated in this study have been deposited in the NCBI Gene Expression Ominibus (GEO) database under the accession number GSE190291. Gene Expression Omnibus. https://www.ncbi.nlm.nih.gov/geo/query/acc.cgi?acc=GSE190291. (The following secure token has been created to allow review of record GSE190291 while it remains in private status: slurgooulfwnbih). To finish the RNA-seq and ATAC-seq data processing, quality control, and mapping to the Sus scrofa genome, only publicly available tools but no other custom code was used. The details about the tools and settings for analyzing RNA-seq data and ATAC-seq can be found on GitHub (https://github.com/1127JJ/Code_availability).
