# Supplementary material for: Transcriptomic and chromatin accessibility dynamics of porcine alveolar macrophages in exposure to fumonisin B1
Source: Front Cell Dev Biol. 2022 Oct 18;10:876247. doi: 10.3389/fcell.2022.876247 (PMC9623295; doi:10.3389/fcell.2022.876247)
Supplement: Supplementary file 1 [file DataSheet1.ZIP › Supplementary Material/Gene Expression Ominibus.docx]

GSE190291      Transcriptomic and chromatin accessibility analyses of porcine
alveolar macrophages exposed to Fumonisin B1  Dec 05, 2023   approved  CSV
XLSX 
GSM5719320     alveolar macrophage, FB1-1, ATAC-seq  Dec 05, 2023   approved 
CSV       
GSM5719321     alveolar macrophage, FB1-2, ATAC-seq  Dec 05, 2023   approved 
CSV       
GSM5719322     alveolar macrophage, FB1-3, ATAC-seq  Dec 05, 2023   approved 
CSV       
GSM5719323     alveolar macrophage, NC-1, ATAC-seq  Dec 05, 2023   approved 
CSV       
GSM5719324     alveolar macrophage, NC-2, ATAC-seq  Dec 05, 2023   approved 
CSV       
GSM5719325     alveolar macrophage, NC-3, ATAC-seq  Dec 05, 2023   approved 
CSV       
GSM5719326     alveolar macrophage, FB1-1, LncRNA-seq  Dec 05, 2023   approved 
None     
GSM5719327     alveolar macrophage, FB1-2, LncRNA-seq  Dec 05, 2023   approved 
None     
GSM5719328     alveolar macrophage, FB1-3, LncRNA-seq  Dec 05, 2023   approved 
None     
GSM5719329     alveolar macrophage, NC-1, LncRNA-seq  Dec 05, 2023   approved 
None     
GSM5719330     alveolar macrophage, NC-2, LncRNA-seq  Dec 05, 2023   approved 
None     
GSM5719331     alveolar macrophage, NC-3, LncRNA-seq  Dec 05, 2023   approved 
None     
GSM5719332     alveolar macrophage, FB1-1, miRNA-seq  Dec 05, 2023   approved 
None     
GSM5719333     alveolar macrophage, FB1-2, miRNA-seq  Dec 05, 2023   approved 
None     
GSM5719334     alveolar macrophage, NC-1, miRNA-seq  Dec 05, 2023   approved 
None     
GSM5719335     alveolar macrophage, NC-2, miRNA-seq  Dec 05, 2023   approved 
None
