# Supplementary material for: Transcriptomic and chromatin accessibility dynamics of porcine alveolar macrophages in exposure to fumonisin B1
Source: Front Cell Dev Biol. 2022 Oct 18;10:876247. doi: 10.3389/fcell.2022.876247 (PMC9623295; doi:10.3389/fcell.2022.876247)
Supplement: Supplementary file 1 [file DataSheet1.ZIP › Supplementary Material/Supplementary Table 1.docx]

| **Group** | **Sample name** | **Sequencing type** | **Target** |
| --- | --- | --- | --- |
| FB1 treatment | FB1_1ln, FB1_2ln, FB1_3ln | rRNA-depleted RNA-seq | lncRNA, mRNA, circRNA |
|  | FB1_1mi, FB1_2mi | miRNA-seq | miRNA |
|  | FB1_1ca, FB1_2ca, FB1_3ca | ATAC-seq | Chromatin accessibility |
| NC | NC_1ln, NC_2ln, NC_3ln | rRNA-depleted RNA-seq | lncRNA, mRNA, circRNA |
|  | NC_1mi, NC_2mi | miRNA-seq | miRNA |
|  | NC_1ca, NC_2ca, NC_3ca | ATAC-seq | Chromatin accessibility |
