# Supplementary material for: Transcriptomic and chromatin accessibility dynamics of porcine alveolar macrophages in exposure to fumonisin B1
Source: Front Cell Dev Biol. 2022 Oct 18;10:876247. doi: 10.3389/fcell.2022.876247 (PMC9623295; doi:10.3389/fcell.2022.876247)
Supplement: Supplementary file 1 [file DataSheet1.ZIP › Supplementary Material/Supplementary Table 2.docx]

| **Sample name** | **Raw reads** | **Clean reads** | **GC content** | **Q20** | **Q30** | **Total Mapped^*^** |
| --- | --- | --- | --- | --- | --- | --- |
| FB1_1ln | 67708842 | 67112752 (99.12%) | 52.41% | 95.58% | 89.30% | 61539903 (92.07%) |
| FB1_2ln | 63458106 | 63003944 (99.28%) | 50.72% | 96.05% | 90.11% | 58251016 (92.75%) |
| FB1_3ln | 72103766 | 71626896 (99.34%) | 50.79% | 96.43% | 90.86% | 65489223 (91.70%) |
| NC_1ln | 73690364 | 73170512 (99.29%) | 51.66% | 96.24% | 90.57% | 68187936 (93.45%) |
| NC_2ln | 67585038 | 67033696 (99.18%) | 52.36% | 96.32% | 90.70% | 62446228 (93.46%) |
| NC_3ln | 65758824 | 65308926 (99.32%) | 51.25% | 95.82% | 89.64% | 60644802 (93.08%) |
