# Supplementary material for: Transcriptomic and chromatin accessibility dynamics of porcine alveolar macrophages in exposure to fumonisin B1
Source: Front Cell Dev Biol. 2022 Oct 18;10:876247. doi: 10.3389/fcell.2022.876247 (PMC9623295; doi:10.3389/fcell.2022.876247)
Supplement: Supplementary file 1 [file DataSheet1.ZIP › Supplementary Material/Supplementary Table 3.docx]

| **Sample name** | **Clean reads** | **High quality^*^** | **Poly A^#^** | **Clean tags^^^** |
| --- | --- | --- | --- | --- |
| FB1_1mi | 9390689 | 9376835 (99.85%) | 1617 (0.0172%) | 9204625 (98.16%) |
| FB1_2mi | 10771428 | 10754578 (99.84%) | 1605 (0.0149%) | 10541321 (98.02%) |
| NC_1mi | 9135939 | 9127049 (99.90%) | 1116 (0.0122%) | 8976449 (98.35%) |
| NC_2mi | 12266061 | 12240460 (99.79%) | 1773 (0.0145%) | 12075820 (98.66%) |
