# Supplementary material for: Transcriptomic and chromatin accessibility dynamics of porcine alveolar macrophages in exposure to fumonisin B1
Source: Front Cell Dev Biol. 2022 Oct 18;10:876247. doi: 10.3389/fcell.2022.876247 (PMC9623295; doi:10.3389/fcell.2022.876247)
Supplement: Supplementary file 1 [file DataSheet1.ZIP › Supplementary Material/Supplementary Table 4.docx]

| **Sample name** | **Raw reads** | **Clean Reads** | **GC content** | **Q20** | **Q30** | **Unique Mapped Reads^*^** |
| --- | --- | --- | --- | --- | --- | --- |
| FB1_1ca | 80004304 | 72831382 (91.03%) | 45.50% | 97.07% | 92.29% | 54227257 (74.46%) |
| FB1_2ca | 86689918 | 77881784 (89.84%) | 46.81% | 97.06% | 92.19% | 55743515 (71.57%) |
| FB1_3ca | 73503494 | 70441804 (95.83%) | 44.75% | 95.92% | 90.07% | 53953353 (76.59%) |
| NC_1ca | 118055924 | 104528362 (88.54%) | 47.50% | 96.64% | 91.17% | 80289273 (76.81%) |
| NC_2ca | 77004768 | 69434288 (90.17%) | 45.78% | 95.42% | 88.47% | 53866958 (77.58%) |
| NC_3ca | 80162686 | 72421842 (90.34%) | 45.65% | 96.83% | 91.60% | 55069712 (76.04%) |
